# The effect of strontium and silicon substituted hydroxyapatite electrochemical coatings on bone ingrowth and osseointegration of selective laser sintered porous metal implants

**DOI:** 10.1371/journal.pone.0227232

**Published:** 2020-01-10

**Authors:** Aadil Mumith, Vee San Cheong, Paul Fromme, Melanie J. Coathup, Gordon W. Blunn

**Affiliations:** 1 Institute of Orthopaedics and Musculoskeletal Science, University College London, Royal National Orthopaedics Hospital, Stanmore, England, United Kingdom; 2 Department of Mechanical Engineering, University College London, London, England, United Kingdom; 3 Department of Automatic Controls and Systems Engineering & Insigneo Institute of *in silico* Medicine, University of Sheffield, Sheffield, England, United Kingdom; 4 College of Medicine, University of Central Florida, Orlando, Florida, United States of America; 5 School of Pharmacy and Biomedical Sciences, University of Portsmouth, Portsmouth, England, United Kingdom; University of Notre Dame, UNITED STATES

## Abstract

Additive manufactured, porous bone implants have the potential to improve osseointegration and reduce failure rates of orthopaedic devices. Substantially porous implants are increasingly used in a number of orthopaedic applications. HA plasma spraying–a line of sight process—cannot coat the inner surfaces of substantially porous structures, whereas electrochemical deposition of calcium phosphate can fully coat the inner surfaces of porous implants for improved bioactivity, but the osseous response of different types of hydroxyapatite (HA) coatings with ionic substitutions has not been evaluated for implants in the same *in vivo* model. In this study, laser sintered Ti6Al4V implants with pore sizes of Ø 700 μm and Ø 1500 μm were electrochemically coated with HA, silicon-substituted HA (SiHA), and strontium-substituted HA (SrHA), and implanted in ovine femoral condylar defects. Implants were retrieved after 6 weeks and histological and histomorphometric evaluation were compared to electrochemically coated implants with uncoated and HA plasma sprayed controls. The HA, SiHA and SrHA coatings had Ca:P, Ca:(P+Si) and (Ca+Sr):P ratios of 1.53, 1.14 and 1.32 respectively. Electrochemically coated implants significantly promoted bone attachment to the implant surfaces of the inner pores and displayed improved osseointegration compared to uncoated scaffolds for both pore sizes (p<0.001), whereas bone ingrowth was restricted to the surface for HA plasma coated or uncoated implants. Electrochemically coated HA implants achieved the highest osseointegration, followed by SrHA coated implants, and both coatings exhibited significantly more bone growth than plasma sprayed groups (p≤0.01 for all 4 cases). SiHA had significantly more osseointegration when compared against the uncoated control, but no significant difference compared with other coatings. There was no significant difference in ingrowth or osseointegration between pore sizes, and the bone-implant-contact was significantly higher in the electrochemical HA than in SiHA or SrHA. These results suggest that osseointegration is insensitive to pore size, whereas surface modification through the presence of an osteoconductive coating plays an important role in improving osseointegration, which may be critically important for extensively porous implants.

## Introduction

Selective laser sintering (SLS) can produce additive manufactured, porous Ti6Al4V structures of complex geometries [1–3]. This allows the manufacture of implants with lower structural stiffness to reduce stress shielding [4,5] and porous structures that mimic bone morphology [6] to optimise bone ingrowth and osseointegration into the implant [1,7] for stable, long-term fixation [3]. Osseointegration improves the implant-stability [2] and load transfer, redistributing forces to the bone more physiologically [8,9], and reduces the risk of fibrous tissue formation [3,5] and implant failure [10,11]. Advances in the fabrication of porous structures using additive manufacturing [12,13] have led to the increased use of SLS implants in cranioplasty, spinal fusion, segmental bone defect, and dental implants, among other orthopaedic applications. The parameters governing bone ingrowth obtained from investigating thin porous coatings onto solid implant surfaces, used for example in hip replacement, may be different from those governing bone ingrowth into substantially porous structures [14]. The design, implant location, stress shielding, bone ingrowth and implant failure may be related, as finite element analysis studies have shown that bone ingrowth into the porous structures can be limited due to stress shielding and that this can increase stress in the implant, possibly reducing the fatigue life of the implant [11]. For these reasons, it is important to optimise bone ingrowth into porous implants, particularly as load bearing structures.

Several studies have investigated the effect of pore size on bone ingrowth into porous implants. Taniguchi et al. [2] examined the use of a diamond lattice design with porosity of 65% in trabecular bone of the rabbit femurs, and three pore sizes of 300, 600 and 900 μm. The 900 μm pore size had the highest ingrowth at 4 weeks, significantly larger than for 300 μm pores. However, at 8 weeks, the 600 μm pore size had the highest ingrowth, but not significantly higher than the other pore sizes. Van der Stok et al. [15] studied the insertion of porous scaffolds with fixed pore size of 490 μm and two different porosities of 68% and 88% in cortical defects of rats and found no significant difference in bone ingrowth. A study on the influence of different microarchitecture with pore sizes between 700 to 1300 μm and open channels between 290 to 700 μm also showed excellent osteoconduction and ingrowth in all cases [16]. A pore size of 1500 μm has also been found to be successful in promoting bone ingrowth in endo-prostheses used for limb reconstruction after bone tumour resection [17]. Taken together, these results suggest that pore sizes of 300–1500 μm are suitable in the design of implants to induce bone ingrowth *in vivo*, while ensuring the development of a vasculature system that is essential for bone formation [18].

Bone ingrowth in porous implants is not sufficient to ensure direct bone-implant fixation as several studies using Tantalum (Ta), Titanium (Ti) and Ti-based alloys have reported the presence of fibrous tissue encapsulating the porous implant [19,20]. One method to improve the bioactivity of bone bonding is through surface modification of these implants, such as through chemical and heat treatment [21]. However, this method is not effective for forming apatite on Ti6Al4V implants, which bonds to both bone and implant surfaces [21], increasing the rate of bone ingrowth and bone-implant contact [1]. Another method is by coating the implants with HA or other calcium phosphate (Ca-P) based coatings through porous surface coatings such as plasma spraying [5], but the line-of-sight technique produces uneven layers of coating and does not coat the complex inner porous structure [4]. Electrochemical deposition of HA provides an alternative method to coat complex geometries [1,4].

Modified coatings and bone graft materials that incorporated various ionic substitutions within stoichiometric HA have been shown to be effective in increasing the bioactivity, thereby encouraging more bone growth or surface osseointegration than HA alone [22–24]. Different anionic and cationic substitutions have been studied, including zinc, fluorine, chlorine, strontium, potassium and silicon, found in natural bone as substituted HA [25]. Various studies involving silicon substituted HA (SiHA) demonstrated an improvement in osteoblastic cell proliferation and morphology [26], adhesion of osteoblasts to the coating [27], and improved bone ingrowth and osseointegration [22,23,28]. Studies that investigated the effect of silicon concentration for *in vitro* and *in vivo* experiments suggested there may be an optimal level of Si for bone formation. Porous bone graft substitutes implanted in the femoral intercondylar notch of rabbits showed that the rate and quality of bone formation and adaptive remodelling were sensitive to the level of Si content in HA [22]. A 0.8 wt% of Si was optimal in achieving the highest amount of bone ingrowth, and higher Si content (1.5 wt%) decreased the mineral apposition rate. This could be related to the cellular response to SiHA as 0.8 wt% Si was demonstrated to upregulate genes for osteoblast development, whereas a 1.6 wt% Si led to reduced proliferation [26].

Strontium (Sr) is also used for substitution within the HA lattice due to its similarity in chemical and physical behaviour to Ca. Moreover, several pre-clinical and clinical studies have demonstrated that strontium ranelate increases bone formation while inhibiting bone resorption [29,30]. Strontium substituted HA (SrHA) coatings have been shown to stimulate osteogenic differentiation of mesenchymal stem cells, while having a negative influence on osteoclasts [31,32]. An optimal Sr content of 3–7 atomic percentage has been suggested [33]. The effect of SrHA with 10 mol % Sr was also shown to improve implant osseointegration and pull-out strength by more than 50% when compared to stoichiometric HA in an osteoporotic rat model [34]. The increase in bone-implant contact was also confirmed in SrHA synthesized using electrochemical methods and implanted in a rabbit model [24].

Several studies have compared SrHA and SiHA in polycaprolactone scaffolds [35] and pure titanium blocks [36]. However, no animal experiments comparing the effects of SrHA and SiHA coatings in Ti6Al4V using electrochemical deposition has been reported, and bone formation within extensively porous coated implants may be different from implants with porous coated surfaces. This study was designed to investigate bone ingrowth into SLS porous structures, electrochemically coated with SiHA and SrHA in an ovine femoral condylar critical sized defect model. The hypothesis is that osseointegration and bone formation in porous implants made from titanium alloy is significantly higher with SiHA and SrHA compared to plasma sprayed or electrochemically coated HA.

## Materials and methods

### Study design

**Fig 1:**
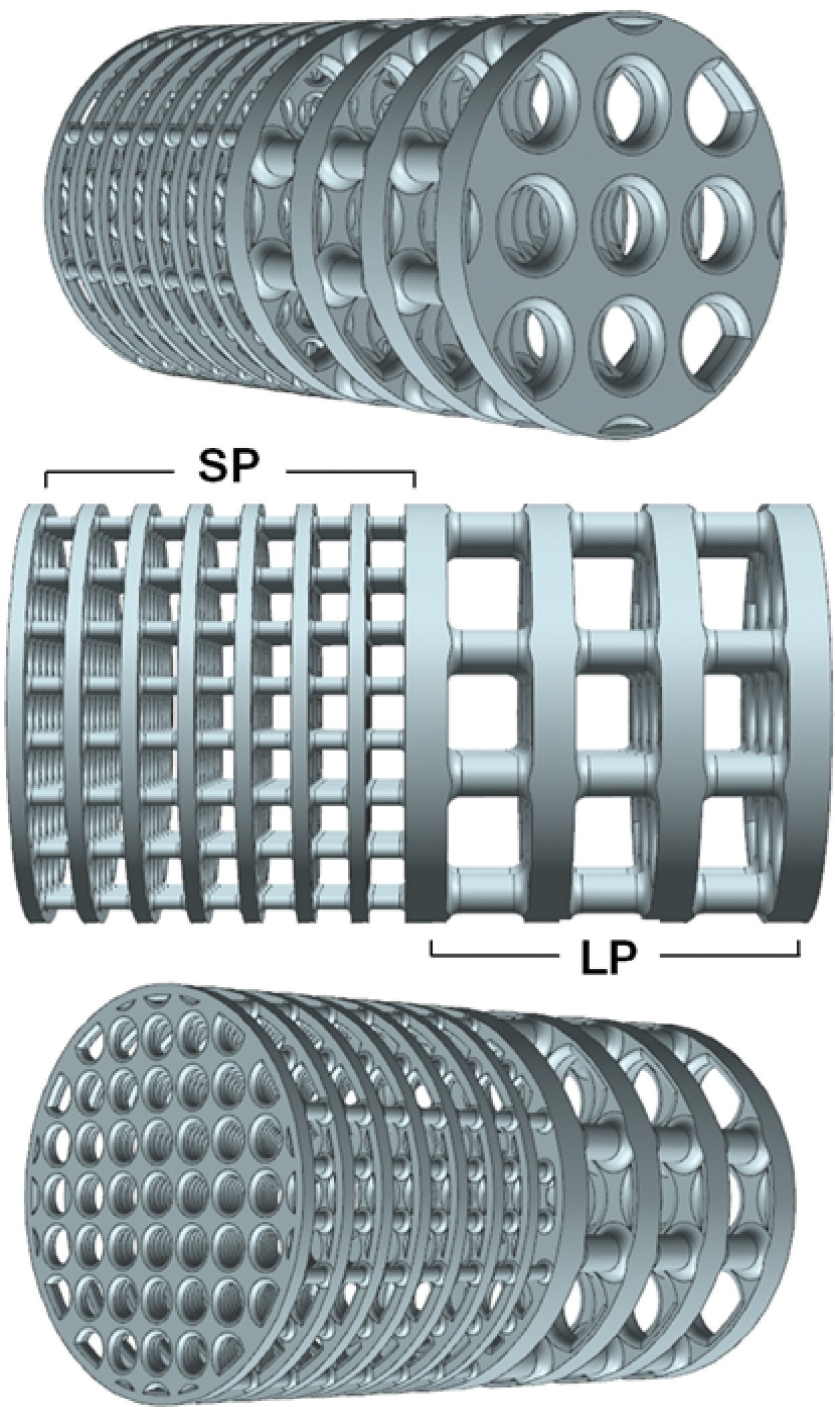
3D models of 700 μm (SP) and 1500 μm (LP) Ti6Al4V implants manufactured as one piece with 8 mm diameter x 14.5 mm length.

Porous cylindrical implants of length 14.5 mm and diameter 8 mm were used in this study [37]. The implant consisted of one half with small pore sizes (SP), 300μm struts and 700μm pore size in cross-section, and the other half with large pores (LP), 750μm struts and 1500μm pore size (Fig 1). The 700 μm and 1500 μm implants were designed with porosities of 75% and 70% respectively, and were manufactured from Ti6Al4V (elastic modulus: 110GPa) using SLS (Eurocoating, Italy). The effective elastic modulus of the two implants, calculated using finite element analysis [11,37], were 18 GPa and 22 GPa for the 700 μm and 1500 μm implants respectively.

The study design consisted of two control groups of uncoated (U) and plasma sprayed (PS) implants (negative and positive control, respectively). The latter were plasma sprayed with a highly crystalline (>85%) hydroxyapatite (Plasma Biotal, UK) that is used routinely for spraying orthopaedic implants. The experimental group comprised of the three different implants—electrochemically coated with HA, SiHA, and SrHA. All implants were randomly allocated to each group.

### Preparation of electrochemical coatings

The uncoated control implants and the implants for electrochemical coating were etched in 2% hydrofluoric acid for 4 minutes. After the acid was neutralised, the implants were ultrasonically cleaned in 10% Decon 90, deionised water, and 99% Industrial Methylated Spirit (IMS) for 15 minutes and left to air-dry prior to being coated.

The electrochemical deposition process used a two-electrode cell configuration. The working electrode (cathode) was the implant while the counter electrode (anode) was a platinum wire 0.063 mm in diameter (Goodfellow Cambridge Ltd, UK). Both electrodes were immersed in an electrolyte with controlled temperature, and stirred gently throughout using a magnetic stirrer. A DC dual power source (Peak Tech, Telonic Instruments Ltd, UK) was used to supply a constant current during the deposition process. The preparation of the electrolytes for the SiHA and SrHA coatings were based on the methods by Huang et al. [38] and Liang et al. [39]. An optimisation process was conducted by varying the duration and current of the electrochemical deposition process to ensure that the thickness of coating formed was even, below the 50 μm threshold described by de Groot et al. [40] and Wang et al. [41], but thick enough to prevent complete removal of the coating during surgery. Ti6Al4V discs of 10 mm diameter and 3 mm thickness were used for the optimisation process. Four discs per coating combination and 16 individual measurements per discs were conducted (S1 Table). Further details on the parameters for the electrochemical deposition are detailed in S1 Data. After electrochemical deposition, all implants were rinsed with deionised water, left to air dry and autoclaved before implantation.

### Coating characterization

Energy dispersive X-ray spectroscopy (EDS) was used to analyse the composition of the coatings on the Ti6Al4V implants (EDX, EDAX Inc, USA). The crystal structures of the coatings were analysed using X-ray diffractometry (XRD), on a Bruker D8 Advance X-ray diffractometer (Brucker, UK). Cu-K_α_ radiation at a 2θ range of 0–100° was used. The phases present were identified by comparing the obtained diffraction patterns with the International Committee for Diffraction Data (ICDD) file cards 9–432 for HA.

### Animal and surgical procedures

All procedures were carried out under Home Office Project Licence 70/8247, and approved by the local Animal Welfare Ethical Review Board, in line with the UK Animal Scientific Procedures Act (1986). All surgery was performed under general anesthesia using isoflurane inhalation (Merial Animal Health Ltd, UK), and all effort was made to minimize suffering. Eight skeletally mature sheep were used in this study. Following general anesthesia, 8x15mm defects were drilled in the medial femoral condyles of adult sheep bilaterally. Implants were pushed into place and the remaining periosteum, fascia and subcutaneous tissues were repaired. The animals recovered in separate pens and once able, were group housed. All animals recovered well and were allowed to weight bear immediately post-operatively. A total of 30 implants, with 6 implants in each group was used in this study. Power analysis based on previous studies indicates that six samples should be used for each group to prove significance with 20% between the mean, a power of 0.8, and a standard deviation of 5%. Four implant positions were possible per sheep (right proximal, right distal, left proximal, left distal) and the position of implants from each group were changed so that they all occupied different positions in the condyles. Furthermore, the implants were also placed with either the smaller 700 μm pore (SP) size oriented inwards (medially) or outwards (laterally). Table 1 outlines the locations and orientation of the implants for each group. The implants were kept *in situ* for 6 weeks, at which point the implants were retrieved for histomorphometric analysis. The animals were euthanized by an overdose of sodium pentobarbital (Merial Animal Health Ltd, UK). To reduce the number of animals in experiments, only one time period was used to to assess the initial bone ingrowth and osseointegration of the implants [11].

**Table 1:** Implant position and orientation within condyle defects. U, uncoated; PS, plasma sprayed HA; HA, electrochemical HA; SiHA, electrochemical SiHA; SrHA, electrochemical SrHA. SP* (700 μm implant) orientated inwards.

	POSITION 1	POSITION 2	POSITION 3	POSITION 4
**1**	U*	PS*	HA*	SiHA*
**2**	SrHA*	U	PS	HA
**3**	SiHA	SrHA	U*	PS*
**4**	HA*	SiHA*	SrHA*	U
**5**	PS	HA	SiHA	SrHA
**6**	U*	PS*	HA*	SiHA*
**7**	SrHA*	U	PS	HA
**8**	SiHA	SrHA		

### Histomorphometry

The retrieved implants were stripped of excess soft tissue and fixed in 10% neutral buffered formalin (NBF) for a minimum period of one week. The specimens were dehydrated in successively increasing concentrations of Industrial methylated Spirit (IMS), de-fatted with 100% chloroform, immersed in 100% IMS to exchange the chloroform and then infiltrated with a 50% IMS and 50% resin mixture (LR White Resin, London Resin Company Ltd, UK), followed by 100% resin. The specimens were embedded in hard grade acrylic resin (LR White Resin, London Resin Company, UK). The resin blocks were sectioned longitudinally through the centre to obtain sections of approximately 80 μm thickness. The thin sections were stained with Toluidine Blue and Paragon to identify soft tissue and bone, and visualised using light microscopy (Axioskop, Carl Zeiss, UK). Histomorphometric analysis was conducted in ImageJ (National Institute of Health, USA) to obtain the bone-to-implant contact (BIC), bone area ratio and soft tissue ratio. BIC was measured as the ratio of the length of bone in direct contact with the implant and the total implant perimeter within the pores. Bone area ratio and soft tissue ratio were computed as the percentage of surface area within the pores occupied by the bone and soft tissue respectively. Thick sections were polished and examined using scanning electron microscopy (JEOL 3500 C, JEOL, Japan). The coating layer identified using SEM was analysed using EDS (EDX, EDAX Inc, USA).

### Statistical analysis

Statistical analysis was performed using SPSS v22 (IBM, USA). As the sample size was small, non-parametric Mann-Whitney U test or Kruskal-Wallis test was used to compare groups.

## Results

### Surface morphology

**Fig 2:**
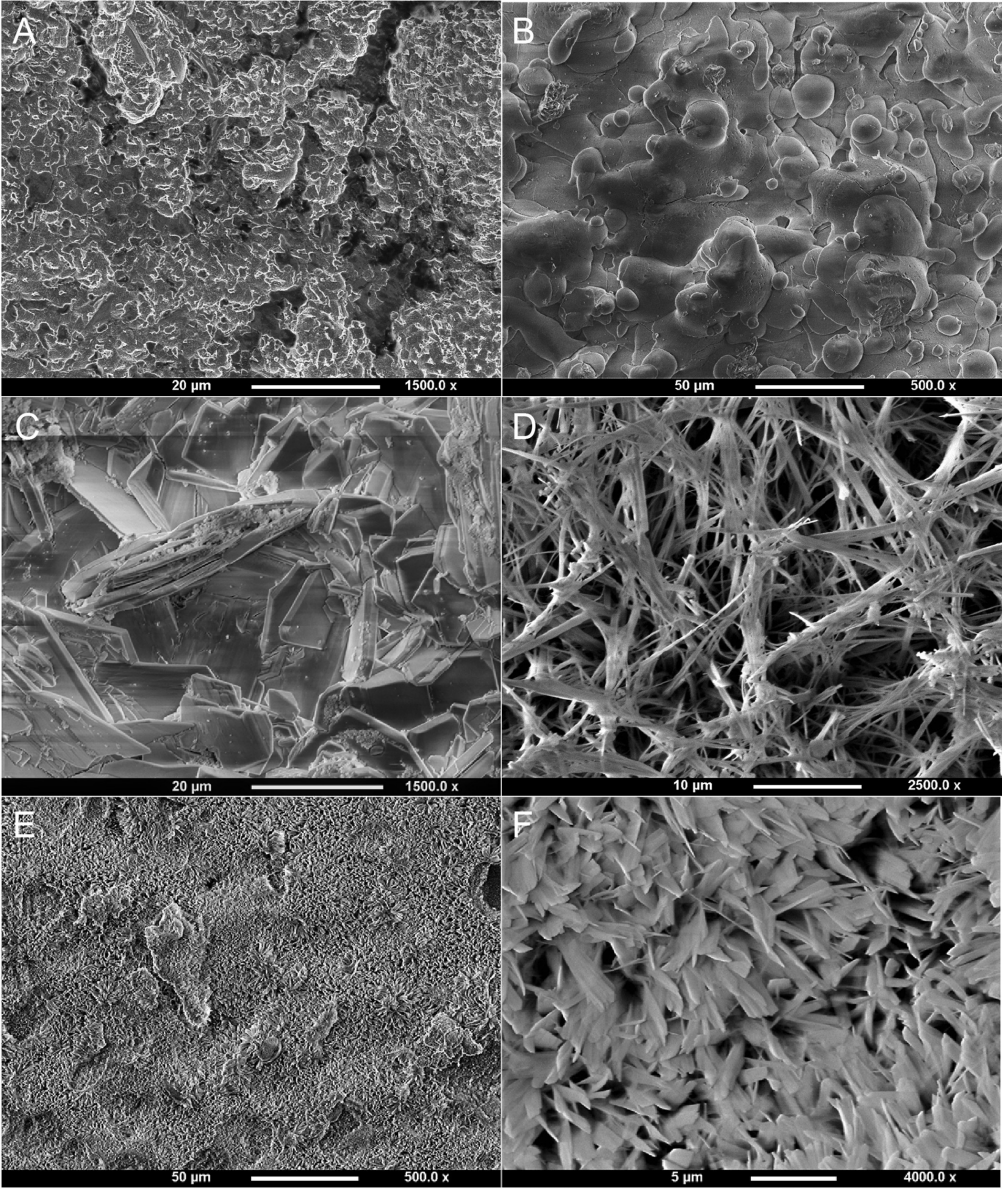
Surface morphology of Ti6Al4V discs with different coatings. (A) Uncoated, roughened, acid-etched. (B) Plasma-sprayed. (C) Electrochemically deposited HA. (D) Electrochemically deposited SiHA. (E-F) Electrochemically deposited SrHA.

SEM examination of Ti6Al4V discs showed differences in the surface morphology depending on the coatings (Fig 2). The uncoated, acid-etched implant had a rough surface with the presence of micro-pits. The plasma-sprayed surface exhibited globular shaped crystallites agglomerated together. Plate-like crystals covered the electrochemically HA and SrHA coated surfaces, but the crystallites were smaller for the SrHA coating. The SiHA coating displayed needle shaped crystals interspersed with plate-like crystals similar to those found in the HA coating, except smaller. For the implant, the electrochemical coatings followed the topology of the pores closely and no loose fragments were seen. None of the pores was blocked by the coating.

### Phase and chemical composition of coating

**Fig 3:**
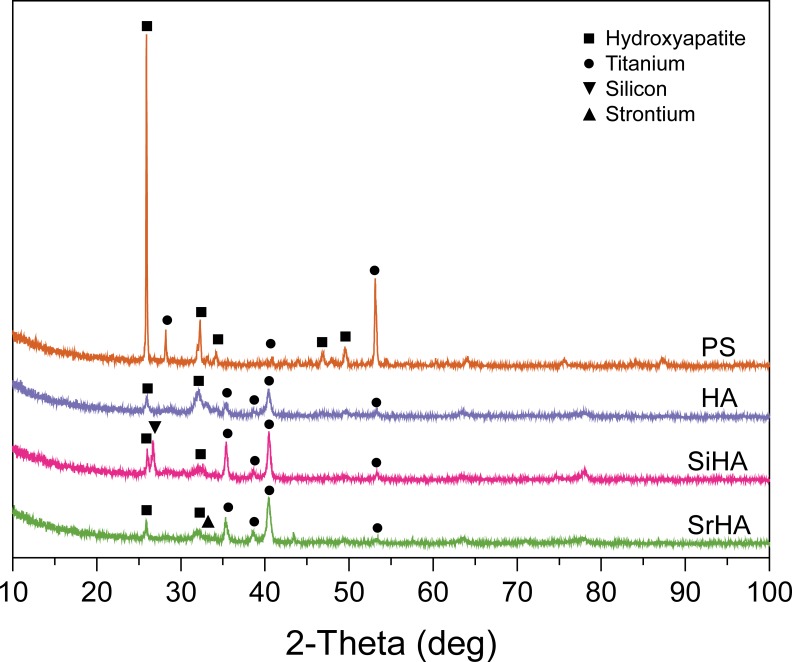
XRD spectra of the coatings on Ti6Al4V structures. PS, plasma sprayed HA; HA, electrochemical HA; SiHA, electrochemical SiHA; SrHA, electrochemical SrHA. ■,●,▼ and ▲ on spectra denote HA, Titanium, Silicon and Strontium peaks respectively.

For the three electrochemical coatings, the molar ratios of the degree of substitution obtained from EDS analysis are summarized in Table 2. The Ca:P ratio of 1.53 ± 0.04 for the HA coating lies between that of tricalcium phosphate (TCP) and stoichiometric HA. The XRD spectra showed that the plasma sprayed HA has the highest level of crystallinity as exhibited by the highest peak (Fig 3). The XRD patterns of the three electrochemical coatings showed lower crystallinity with amorphous phases. Among the substituted-HA coatings, the peak of Sr was lower than that of Si, suggesting that Sr was less incorporated in a crystalline structure than Si in the coatings.

**Table 2:** Elemental analysis of coatings with the appropriate Ca:P ratio and wt% of substituted element shown.

Coating	Ratio (±SD)		wt% (±SD)
HA	Ca:P	1.53±0.04	
SiHA	Ca:(P+Si)	1.13±0.07	Si 1.63±0.81
SrHA	(Ca+Sr):P	1.31±0.03	Sr 4.08±0.06

### Histological observations

**Fig 4:**
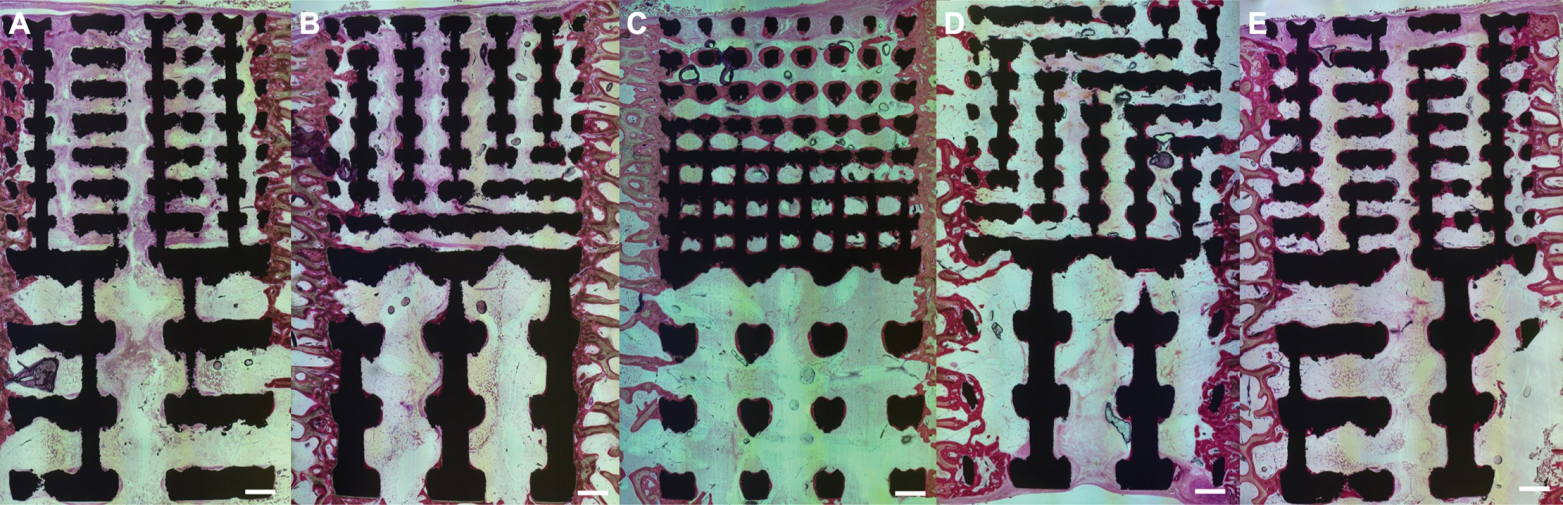
Light microscopy images of histological slices of implants retrieved from ovine femoral condylar defect after 6 weeks: (a) uncoated, (b) plasma sprayed HA, (c) electrochemical HA, (d) electrochemical SiHA and (e) electrochemical SrHA. Toluidine blue and Paragon used to stain bone and soft tissues pink and purple respectively. Black regions correspond to locations of the Ti6Al4V struts. Scale bar indicates 1mm.

After retrieval at 6 weeks, all coatings were still present on the Ti6Al4V surfaces (Fig 4). The plasma sprayed HA coating did not fully cover the inner pores of the porous scaffolds, with increased but incomplete penetration for the larger 1500 μm pore size compared to the smaller 700 μm size structure. The electrochemical coatings completely covered the outer and inner porous surfaces. For all implants and coatings, bone appeared to have grown directly from the surrounding cancellous and cortical bone into the porous implant (Fig 4). Bone formation was highest at the outer regions, at the interface with surrounding bone, and reduced towards the center of the porous implant. The majority of the pores were only partially filled with bone. Osseointegration was observed in all implants at the outer regions, with no observable difference in the level of osseointegration between the two pore sizes. A thin layer of bone covering the inner struts of the porous structure was observed only for implants with one of the three electrochemical coating.

**Fig 5:**
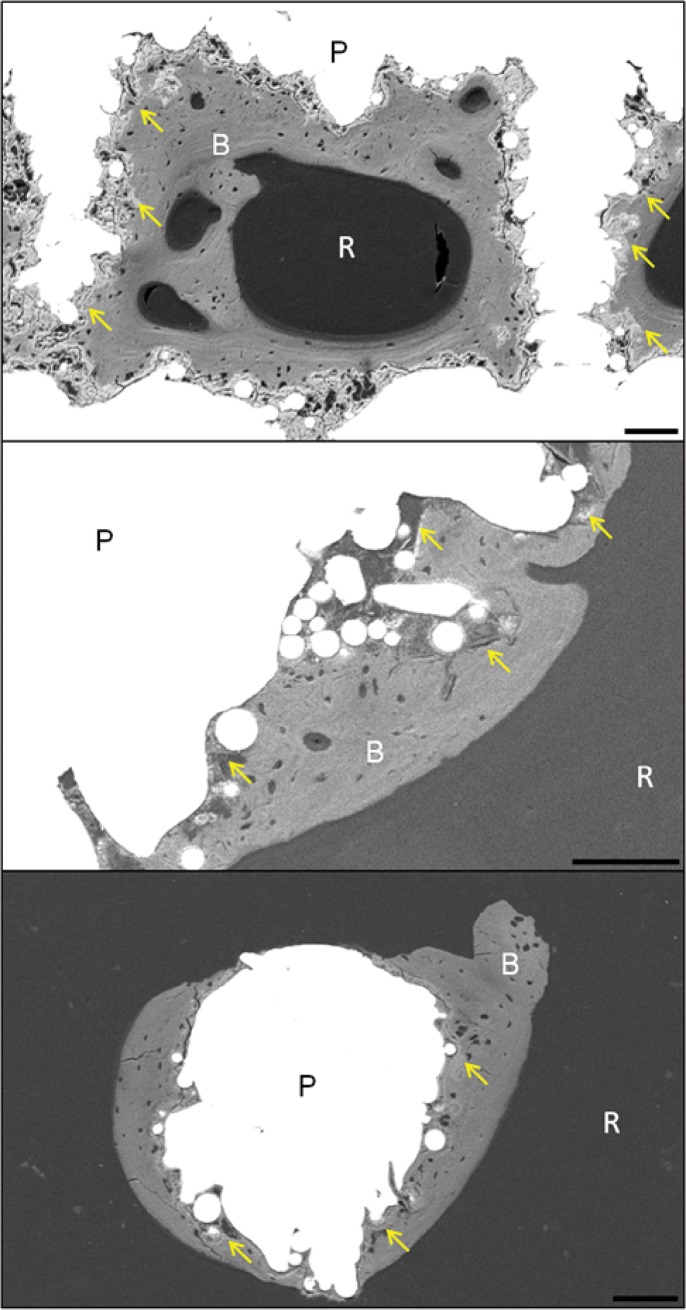
**Backscattered scanning electron microscopy (SEM) images of the implant cross-sections for (top) electrochemical HA (x100), (middle) electrochemical SiHA (x200) and (bottom) electrochemical SrHA (x120) coatings.** Scale bars indicate 100 μm. Yellow, blue and red arrows identify presence of coatings, Haversian canals and lacuna respectively. P: struts. R: soft tissue. B: bone.

SEM images of the implants revealed that all the coating were still present at 6 weeks (Fig 5). There was direct bone attachment with the coatings, and at 6 weeks the bone formed was lamellar (Fig 5). Bone growth was observed adjacent to the coatings, on both struts and pores, even within the inner regions of the porous implants (Fig 4). No isolated islands of bone formation were observed within any of the pores, nor were any of the pores completely filled with bone. Within the inner pores, bone formation was entirely associated with electrochemical coating.

### Histomorphometric analysis

**Fig 6:**
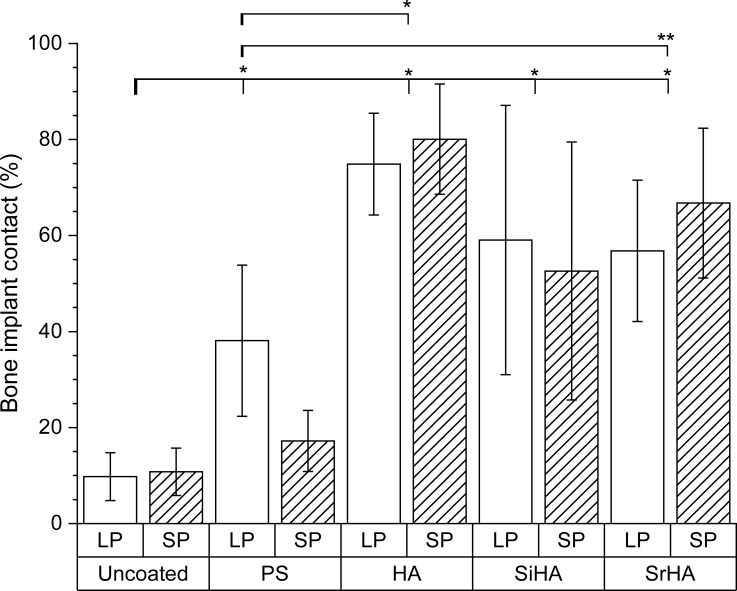
Extent of osseointegration measured by bone implant contact. Boxes indicate mean and lines standard deviations. PS, plasma sprayed HA; HA, electrochemical HA; SiHA, electrochemical SiHA; SrHA, electrochemical SrHA; SP, 700 μm implant; LP, 1500 μm implant. *p < 0.001, **p < 0.02.

The total implant surface length within the pores for the implants with the 700 μm and 1500 μm pores were 149.3 ± 11.6 mm and 69.4 ± 7.1 mm (mean ± S.D.) respectively, and this was significantly larger for the implant with the smaller pores than the implant with the larger pores (p<0.001). The BIC in implants with the smaller and larger pore size were 47.7 ± 27.4% and 45.5 ± 31.1% (mean ± S.D.) respectively, not significantly different (p = 0.853). There was no significant difference in the BIC between the two pore sizes for all 5 types of coating considered individually (p = 0.811, 0.066, 0.378, 0.936 and 0.298 for uncoated, plasma-sprayed, HA, SiHA and SrHA respectively). Therefore, the results for both pore sizes were combined together to test the hypothesis. For the plasma sprayed group, only the results from the larger pore size were considered in further analysis, as there was a marked difference in BIC (Fig 6), potentially due to the incomplete coating coverage of the inner pores, especially for the smaller 700 μm size structure, thereby giving a statistical result that was tending towards significance (p = 0.066).

Using the Kruskal-Wallis test, there was a significant difference in BIC when all 10 coating-pore size combinations were analysed (p<0.001) (Fig 6). The plasma-sprayed, electrochemical HA, SiHA and SrHA of both pore sizes had BIC of 38.1 ± 15.7%, 77.5 ± 10.9%, 55.8 ± 26.4% and 61.8 ± 15.4% respectively, which were significantly larger than the BIC of 10.3 ± 4.8% in the uncoated control (p<0.0001 for all cases). Comparing the electrochemical coating against the plasma-sprayed implants with the larger pore size, the BIC were significantly larger than plasma sprayed HA for electrochemically deposited HA (p<0.001), and SrHA (p = 0.01) but not for SiHA (p = 0.1627). The BIC in SiHA and SrHA were both not significantly different to the BIC for electrochemical deposited HA (p = 0.973 and p = 0.987 respectively). Table 3 summarises the significant results for the individual cases, and showed that significant results were obtained which supported the first hypothesis, the comparison to the plasma sprayed implants as positive control.

**Table 3:** p-values and percentage BIC (mean ± S.D.) for coating compared against uncoated, plasma spray coated, and electrochemical HA implants. **Implants from both pore sizes were grouped together (plasma sprayed only results from larger pore size).** U, uncoated PS, plasma sprayed HA; HA, electrochemical HA; SiHA, electrochemical SiHA; SrHA, electrochemical SrHA.

Implant *(% integration ± S*.*D*.*)*	PS (LP only) *(38*.*1 ± 15*.*7)*	HA *(77*.*5 ± 10*.*9)*	SiHA *(55*.*8 ± 26*.*4)*	SrHA (61.8 *±* 15.4)
**U** *(10*.*3 ± 4*.*8)*	<0.001	<0.001	<0.001	<0.001
**PS** *(38*.*1 ± 15*.*7)*		<0.001	0.1627	0.01088
**HA** *(77*.*5 ± 10*.*9)*			0.97345	0.98689

Evaluation of bone formation (bone within the pores) revealed that the area differences were very small. No statistically significant difference was found between the implants with the larger pore size. For the implants with the 700 μm pores, the bone area ratios were significantly higher only in the electrochemical coated HA and SrHA implants compared with the negative control (p = 0.007, 0.0464 respectively). None of the electrochemically coated implants had significantly higher bone area ratio than the plasma sprayed positive control, and the bone area ratio for SrHA and SiHA were not significantly higher than for the electrochemical HA implant.

## Discussion

In this study, implants with pore sizes of 700 μm and 1500 μm were electrochemically coated with HA, SiHA and SrHA, and inserted into an ovine condylar defect model for 6 weeks. The effect on bone ingrowth and osseointegration against uncoated and plasma-sprayed implants was compared. Although the performance of SiHA and SrHA had been investigated in polycaprolactone scaffolds and pure titanium blocks [35,36], this is the first study that directly compared the *in vivo* osseoconductivity and ingrowth of three electrochemical coatings in laser-sintered Ti6Al4V implants of two pore sizes, against two controls. Results showed that bone formation occurred along the metallic structure and not in the interior pores. There was high osseointegration of over 50% BIC for the electrochemically coated implants, significantly higher than the plasma sprayed implants (positive control) for HA and SrHA, but there was no significant increase in the bone area ratio in any of the electrochemically coated implants compared to the plasma sprayed implants.

Electrochemical deposition offers the advantage of fully coating the interior surfaces of porous implants, over line-of-sight processes such as plasma spraying, which can only coat the outer regions. It has also been shown in literature that substituting calcium with strontium and silicon can enhance bone formation compared to plain HA coating. This led to the hypothesis that electrochemically coated SiHA and SrHA implants would have significantly higher osseointegration and bone ingrowth than plasma sprayed or electrochemically coated HA implants. Substituted hydroxyapatites are thought to provide a more physiological mineral, as HA found in native bone contains many ionic substitutions. Several studies have demonstrated the potential of SiHA and SrHA coatings to increase the reactivity of osteoblast and bone apposition rate over unsubstituted HA [22,24,34].

The surface morphologies were different for all the coatings in this study; electrochemically coated HA and SrHA both displayed plate-like crystalline structures, which were smaller in scale in SrHA than HA. SiHA had needle-like crystalline structures and plasma-sprayed coating was present as HA globules. XRD analysis revealed that plasma sprayed coating was the most crystalline, while the electrochemical coatings had similar XRD spectra, with amorphous phase present. Highly crystalline structures have improved coating longevity, due to reduced solubility compared to amorphous ones [34]. However, bone mineral has lower crystallinity compared to many commercial plasma sprayed HA coatings. The coatings in this study were present at 6 weeks in the animal model, and the high BIC of above 50% for the electrochemically coated implants of both pore sizes, and above 35% for plasma-sprayed implants with the larger pores suggest that implant fixation had occurred. Within the porous structure, bone occurred only on electrochemically coated surfaces. The lumen of the pores were not filled, suggesting that the bone response was associated with the osteoconductivty of the surface. Using the same animal model, Reznikov et al. [42] noted that bone regeneration within the interior of the scaffold was inversely proportional to scaffold stiffness and was strain driven. This is in line with the theoretical work carried out by Cheong et al. [37] who used adaptive finite element algorithms to predict bone ingrowth in additive manufactured porous implants and showed that stress shielding of porous implants made from conventional titanium alloy limited ingrowth and osseointegration, reducing the stimulus for bone remodeling in the inner pores below the minimum level for bone formation for both pore sizes. This would explain the observation in this study that coating had less effect on overall bone formation than surface integration (BIC). The finite element analysis also showed that the presence of coating has a similar effect to lowering the stimulus for bone formation, leading to improved bone ingrowth. These results suggest that surface modification of porous scaffolds that fully coats all the porous regions of the implant is influential in encouraging osseointegration, but overall bone ingrowth depends to a larger degree on the structural stiffness and stress shielding within the implant.

The results in our current study showed improved osseointegration of electrochemical HA, SiHA and SrHA, over the negative, uncoated control. Electrochemical HA and SrHA also had significantly higher BIC over the positive control of plasma-sprayed HA. This could be due to the increased solubility of the amorphous phase in the electrochemical coatings, as the dissolution of HA accelerates the formation of biological apatite to bond with bone tissues [24,43]. The lower BIC in the smaller pore, plasma-sprayed implant is likely due to the coating not reaching the inner regions of the porous implant as it is a line-of-sight technique.

The results showed no statistically significant difference between the osseointegration (BIC) among the three electrochemical coatings. Surface morphology is known to affect cell attachment and differentiation of osteoblasts [44], and possible reasons for this finding could be due to the similarity in surface roughness of these three coatings (4–5 μm (Ra), not statistically different) (S2 Table). However, the control implants were also etched and had the same degree of surface roughness, and there was no difference in the proliferation or the differentiation of these cells on electrochemically coated surfaces with the uncoated control *in vitro* (S3 and S4 Tables). Hence, this effect is more likely associated with the chemical nature of the coatings in a 3D structure.

The second reason could be due to the composition of the substituted element. The SiHA coating in this study had a 1.63wt% of Si, which is similar to the results presented by Hing et al. [22], which showed in a rabbit model that bone graft substituted with 1.5wt% Si had no significant difference in bone ratio compared to 0wt% Si in bone ratio at 1, 3, 6 and 12 weeks. A 7.7wt% of Si has been reported to significantly increase MC3T3-E1 osteoblast cell growth in an *in vitro* study [38], but the optimum level found for bone formation in an *in vivo* study is 0.8wt% [22]. Higher levels of Si have been linked with increased cytotoxicity [45], which accumulates faster in 3D porous structures as waste removal is slower than on 2D surfaces. Moreover, if Si substitution increases above 2 wt%, HA starts to destabilise and alpha triclacium phosphate is formed [46].

The SrHA in this study utilized a Sr/(Sr+Ca) ratio of 10%, but the results obtained are different from literature utilizing the same molar ratio of Sr for the sol-gel-dip coating method, where significantly higher bone formation was demonstrated compared with non-substituted controls in rabbit and mouse models [24,34]. The reference methodology for SrHA coating reported a measured Sr content of 4.7 wt%, which is marginally higher than the 4.08 wt% used in this study [39]. The authors also reported significantly improved osteoblast proliferation *in vitro* and osseointegration around the threads of implant screws, compared to 5% SrHA and uncoated implants [39]. In our study, SrHA did not perform better than the other electrochemically deposited coatings and this could be due to stress shielding within the pores. Sr concentrations of 6.25 atomic percentage (at%) has been reported to significantly increase both bone area ratio and BIC in porous implants, but the measured value in this study was only 1%. The Ca:P ratio of 1.31 obtained in this study is also higher than the value of 1.1 that Liang et al. [39] obtained, suggesting that the SrHA coating obtained has a closer chemical resemblance to stoichiometric HA.

The implants were designed with pore sizes of 700 μm and 1500 μm, within the range of pore sizes that have reported good bone ingrowth *in vivo*. The implants had porosities of 75% and 70% respectively, leading to a larger internal surface area for bone attachment in the smaller pore size. No significant difference for BIC between the two pore sizes was found in line with literature [2,15].

This study has several limitations. Firstly, the femoral condyle defect model utilised in this study may not be fully representative of the mechanical forces encountered in functional implants used in humans. The lower forces in the trabecular bone surrounding the implant may be the reason for the relatively low amount of bone ingrowth compared with porous implants in canine diaphysis with above 50% of bone ingrowth reported [47]. The extent of bone ingrowth was only assessed at one time point, at 6 weeks, which may not be at equilibrium [37]. Although this duration is commonly used in small ruminants to assess the initial success of implants [19,42], further testing at multiple time points would be required to assess the long-term performance of the implant. Long term testing may also be important as previous studies in goats have shown the resorption of apatite coating not in direct contact with the implant and newly formed bone, after 6 weeks [19,48]. FEA studies using new algorithms for osteoconduction, verified with histology results, showed that the presence of coating lowers the mechanical stimulus for bone formation [11,37]. Hence, the dissolution of the coating may affect the mechanical stimulus required to maintain the bone. The histological analysis was conducted with the aim of evaluating bone ingrowth and osseointegration and used only one type of staining due to the limited sample size. Future studies could also include other staining methods such as Haemotoxylin and Eosin to better understand the process of osteogenesis, or fluorochrome labelling to investigate bone apposition and the rate of osteoconduction. Nevertheless, this study highlights the importance of coating the inner pores of substantially porous structures. This study used different conditions to produce the coatings in order to maximise bone ingrowth. The results showed that further work to develop the electrochemical deposition process is required to lower the Si content and increase the Sr content to optimum levels reported for sol-gel-dip coating. Wettability studies have not been conducted in this study as HA is osteoconductive, and the low amount of substitution was not expected to change the surface energy of the coatings significantly. However, this could be considered as part of future work to determine the effect of substitution on the surface energy of the coatings. This study only investigated the *in vivo* response of implants with two pore sizes with similar porosities. The results in this paper showed that there is a significant difference in BIC and bone ratio between implants that are HA-coated or uncoated, but porous implants with porosities of 63% and 49% manufactured by EBM and SLM have both shown no significant difference whether the implants are uncoated or HA-coated in a goat model [1]. The link between stress shielding and osteoconductivity and bone ingrowth should thus be considered in the development and design of implants for human use.

## Conclusions

This study compared the extent of bone ingrowth and osseointegration of completely porous implants with electrochemically deposited HA coating with three different ionic substitutions, in an *in vivo* model for the first time. The results indicated that unsubstituted HA and SrHA led to significantly increased osseointegration compared to uncoated and HA plasma sprayed control groups, whereas there was no significant difference between the pore sizes tested. This suggests that for porous implants, osseointegration is less driven by pore size than by the presence of a bioactive coating and the overall stiffness of the implant. For implants electrochemically coated with HA, over 80% of the inner porous surface was osseointegrated, whereas osseointegration was about 10% with non-coated implants in the outer pores. The combination of porous structures with electrochemically deposited coating has the potential to be developed further as a bone ingrowth region in segmental prostheses, to protect the implant from failure.

## Supporting information

S1 Data Experimental details on the electrochemical deposition of HA, SiHA and SrHA. (PDF)

S1 Table Optimisation of the parameters for electrochemical deposition of SiHA and SrHA using 10 mm diameter and 3 mm thickness discs. (PDF)

S2 Table Summary of surface roughness (Ra; nm) measured from 10 mm diameter and 3mm thickness discs. (PDF)

S3 Table Quantification of ovine mesenchymal stem cells (MSCs) using AlamarBlue assay. (PDF)

S4 Table Quantification of osteogenic differentiation of ovine mesenchymal stem cells (MSCs) for all coatings on 10 mm diameter and 3mm thickness discs. (PDF)
