# Supplementary material for: The effect of strontium and silicon substituted hydroxyapatite electrochemical coatings on bone ingrowth and osseointegration of selective laser sintered porous metal implants
Source: PLoS One. 2020 Jan 10;15(1):e0227232. doi: 10.1371/journal.pone.0227232 (PMC6953817; doi:10.1371/journal.pone.0227232)
Supplement: S1 Data — (PDF) [file pone.0227232.s001.pdf]

The effect of strontium and silicon substituted hydroxyapatite electrochemical coatings on bone ingrowth and osseointegration of selective laser sintered porous metal implants

Aadil Mumith, Vee San Cheong, Paul Fromme, Melanie J. Coathup, Gordon W. Blunn

Supporting information

S1 Supplementary Data

Electrochemical HA

Analytical grade $Ca(H_2PO_4)_2$ (Sigma-Aldrich, UK) was dissolved in deionised water to form 0.13M calcium phosphate solution. Deposition was conducted by passing a 130mA current at room temperature for 15 minutes. Subsequently, the implants were immersed in 0.1M NaOH solution for 72 hours to convert the initial calcium phosphate precipitate (Ca_2HPO_4-$2H_2O$; Brushite) into hydroxyapatite, $Ca_5(PO_4)_3OH$.

Electrochemical SiHA

The electrolyte contained 1g/l of nano-SiO_2 (Sigma-Aldrich), 0.042M $Ca(NO_3)_2.4H_2O$ (Sigma-Aldrich, UK), and 0.025M $NH_4H_2PO_4$ (Sigma-Aldrich, UK) in $^dH_20$. The supersaturated electrolyte was adjusted to pH 4.2 using 10% HCl and 10% NaOH (Fisher Scientific, UK) at room temperature. Before electrochemical deposition, the implants were anodized by submerging them in 5M NaOH solution at 65°C whilst being actively stirred. Due to the relative non-reactivity of Ti6Al4V to alkali treatment, this process was done for 24 hours instead of 12 hours for commercially pure Ti.

A deposition time of 45 minutes, using a current density of 0.8 mA $/cm^2$ was found to give the thickest coating of 19.2 ± 5.7 μm (mean ± SD) for the optimisation process. Hence the porous implants were coated using a current of 11.2mA for 45 minutes at 65°C. Once coated, the implants were placed in a RHF 1600 furnace (Carbolite Gero, UK) at 300°C for 2 hours.

Electrochemical SrHA

The effect of strontium and silicon substituted hydroxyapatite electrochemical coatings on bone ingrowth and osseointegration of selective laser sintered porous metal implants

Aadil Mumith, Vee San Cheong, Paul Fromme, Melanie J. Coathup, Gordon W. Blunn

The electrolyte contained 0.06M $CaCl_2$, 0.036M $NH_4H_2PO_4$, 0.00667M of $SrCl_2$ (Sigma-Aldrich, UK) and 0.2M NaCl (S7653; Sigma-Aldrich, UK) in ^dH_2O. The $SrCl_2$ acted as the Sr source with the NaCl as the conducting agent. The solution was adjusted to a pH of 4.5 by the addition of 10% HCl (Fisher Scientific, UK) at room temperature.

An optimisation process was conducted using Ti6Al4V discs by varying the duration and current of the coating process. A current density of 15 mA /cm^2 and a deposition time of 2.5 minutes gave the most even coating. The thickness of 14.2 ± 5.2 μm (mean ± SD) was also closest to the thickness obtained for SiHA (S1 Table). Therefore, the porous implants were coated using a current of 210mA over 2.5 minutes at 60°C. After coating, the implants were placed in 0.1M NaOH for 72 hours at room temperature before being rinsed with deionised water and left to air dry.
