# Supplementary material for: The effect of strontium and silicon substituted hydroxyapatite electrochemical coatings on bone ingrowth and osseointegration of selective laser sintered porous metal implants
Source: PLoS One. 2020 Jan 10;15(1):e0227232. doi: 10.1371/journal.pone.0227232 (PMC6953817; doi:10.1371/journal.pone.0227232)
Supplement: S1 Table — (PDF) [file pone.0227232.s002.pdf]

**S1 Table.** Optimisation of the parameters for electrochemical deposition of SiHA and SrHA using 10 mm diameter and 3 mm thickness discs.

| Coating | Current density ($mA/cm^2$) | Deposition time (minutes) | Thickness (μm±SD) |
|---|---|---|---|
| SiHA | 0.8 | 30 | 9.9±2.7 |
| | 0.8 | 45 | 19.2±5.7 |
| SrHA | 7.5 | 2.5 | 6.9±1.4 |
| | 7.5 | 5 | 14.8±2 |
| | 15 | 2.5 | 14.2±5.2 |
| | 15 | 5 | 55.6±21 |
