# Supplementary material for: The effect of strontium and silicon substituted hydroxyapatite electrochemical coatings on bone ingrowth and osseointegration of selective laser sintered porous metal implants
Source: PLoS One. 2020 Jan 10;15(1):e0227232. doi: 10.1371/journal.pone.0227232 (PMC6953817; doi:10.1371/journal.pone.0227232)
Supplement: S2 Table — (PDF) [file pone.0227232.s003.pdf]

S2 Table. Summary of surface roughness (Ra; nm) measured from 10 mm diameter and 3mm thickness discs.

Coating	Ra (±SD)
Uncoated	2619.65±460.54
PS HA	3123.53±232.23
EHA	4192.41±405.66
ESiHA	4387.80±303.98
ESrHA	3881.97±76.37
