# Supplementary material for: The effect of strontium and silicon substituted hydroxyapatite electrochemical coatings on bone ingrowth and osseointegration of selective laser sintered porous metal implants
Source: PLoS One. 2020 Jan 10;15(1):e0227232. doi: 10.1371/journal.pone.0227232 (PMC6953817; doi:10.1371/journal.pone.0227232)
Supplement: S3 Table — (PDF) [file pone.0227232.s004.pdf]

S3 Table. Quantification of ovine mesenchymal stem cells (MSCs) using AlamarBlue assay for all coatings on 10 mm diameter and 3mm thickness discs.

Time point (days)	Coating	Normalised AlamarBlue assay (±SD)
3	Uncoated	104.3±2.5
	Plasma sprayed	105.9±3.0
	HA	80.4±1.5
	SiHA	87.5±6.4
	SrHA	101.2±4.2
7	Uncoated	117.5±18.1
	Plasma sprayed	125±26.1
	HA	96.8±19.2
	SiHA	99.9±8.4
	SrHA	126.8±41.7
14	Uncoated	160.5±95.3
	Plasma sprayed	184.9±64.5
	HA	164.1±44.2
	SiHA	86.9±44.7
	SrHA	237.9±16.8
