# Supplementary material for: The effect of strontium and silicon substituted hydroxyapatite electrochemical coatings on bone ingrowth and osseointegration of selective laser sintered porous metal implants
Source: PLoS One. 2020 Jan 10;15(1):e0227232. doi: 10.1371/journal.pone.0227232 (PMC6953817; doi:10.1371/journal.pone.0227232)
Supplement: S4 Table — (PDF) [file pone.0227232.s005.pdf]

S4 Table. Quantification of osteogenic differentiation of ovine mesenchymal stem cells (MSCs) for all coatings on 10 mm diameter and 3mm thickness discs.

Timepoint (days)	Coating	Normalised ALP assay (±SD)
3	Uncoated	10.1±3.4
	Plasma sprayed	8.3±1.6
	HA	5.7±1.7
	SiHA	9.3±2.7
	SrHA	8.1±1.7
7	Uncoated	8.7±2.8
	Plasma sprayed	7.2±1.8
	HA	9.2±1.4
	SiHA	8.8±4.3
	SrHA	6.9±0.9
14	Uncoated	12.6±5.2
	Plasma sprayed	10.1±5.4
	HA	7.8±0.6
	SiHA	9.9±5.8
	SrHA	12.6±3.1
